# 5-Aminolevulinic Acid-Mediated Photodynamic Therapy Potentiates the Effectiveness of Doxorubicin in Ewing Sarcomas

**DOI:** 10.3390/biomedicines10112900

**Published:** 2022-11-11

**Authors:** Lea Marocco, Felix Umrath, Saskia Sachsenmaier, Robert Rabiner, Nikolaus Wülker, Marina Danalache

**Affiliations:** 1 Laboratory of Cell Biology, Department of Orthopaedic Surgery, University Hospital of Tübingen, 72072 Tübingen, Germany; 2 Department of Oral and Maxillofacial Surgery, University Hospital of Tübingen, 72076 Tübingen, Germany; 3 Department of Orthopaedic Surgery, University Hospital of Tübingen, 72076 Tübingen, Germany; 4 IlluminOss Medical Inc., East Providence, RI 02914, USA

**Keywords:** Ewing sarcoma, photodynamic therapy, doxorubicin, combination therapy, cytoskeleton, atomic force microscopy

## Abstract

Ewing sarcomas (ES) are aggressive primary bone tumors that require radical therapy. Promising low toxicity, 5-aminolevulinic acid (5-ALA)-mediated photodynamic therapy (PDT) could enhance the effectiveness of conventional treatment modalities (e.g., doxorubicin (DOX)), improving, thus, the anti-tumorigenic effects. In this study, we investigated the effects of DOX and 5-ALA PDT alone or in combination on three different human ES cell lines. Cell viability, reactive oxygen species (ROS) production, and cellular stiffness were measured 24 h after PDT (blue light-wavelength 436 nm with 5-ALA) with or without DOX. ES cell lines have a different sensitivity to the same doses and exposure of 5-ALA PDT. DOX in combination with 5-ALA PDT was found to be effective in impairing the viability of all ES cells while also increasing cytotoxic activity by high ROS production. The stiffness of the ES cells increased significantly (*p* < 0.05) post treatment. Overall, our results showed that across multiple ES cell lines, 5-ALA PDT can successfully and safely be combined with DOX to potentiate the therapeutic effect. The 5-ALA PDT has the potential to be a highly effective treatment when used alone or in conjunction with other treatments. More research is needed to assess the effectiveness of 5-ALA PDT in in vivo settings.

## 1. Introduction

Ewing sarcoma (ES) is a group of highly malignant tumors that primarily affect the bones and soft tissue of children and young adults, making it the second most common of its kind [1,2]. While ES occurs in any part of the body and can cause severe pain and local swelling, it is usually not detected until this stage [3,4]. Risk-adapted therapy is essential due to the high mortality caused by early metastases and includes neoadjuvant or adjuvant chemotherapy, local removal, and radiotherapy [5,6,7]. In this conventional approach, induction chemotherapy has prevailed, which consists of, among other drugs, the anthracycline doxorubicin (DOX) [2]. Although DOX is characterized by multiple mechanisms of cytotoxic action, including: DNA intercalation; topoisomerase II poisoning; generation of free radicals and oxidative stress; and membrane damage via altered sphingolipid metabolism [8], it has limited therapeutic use due to severe side effects that cause toxicity to organs such as the heart, brain, liver, and kidney [9,10]. Subsequent radical surgery and radiation can raise the chances of recovery for patients without metastases to 65%, however, the 3-year survival rate for already metastatic findings remains at only 25% [11]. New, safe, and effective treatment modalities that complement conventional approaches are desperately needed in the complex landscape of ES. Due to its potential for treating both neoplastic and non-neoplastic diseases, photodynamic therapy (PDT) has been the focus of numerous research efforts in recent years [12,13]. Put simply, PDT uses so-called photosensitizers (PS) to damage target tissue by harnessing the energy of light [14,15]. The otherwise harmless PS can produce reactive oxygen species (ROS) from molecular oxygen when they are excited by certain wavelengths. The highly reactive ROS that are produced when a cell is exposed to light excitation damage the cell by attacking various cellular components such as the cell membrane or proteins, leading to cell death [16]. As a PS, 5-aminolevulinic acid (5-ALA) has been approved by both the Food and Drug Administration and the European Medicine Agency due to its low toxicity and rapid metabolization [17]. Because 5-ALA is an inactive precursor in the heme pathway, as opposed to earlier PS, the risk of negative phototoxic effects that plagued its predecessors was greatly diminished [18]. Exogenously administered 5-ALA is metabolized into protoporphyrin IX (PpIX). Because the activity of key enzymes in tumor cells is significantly altered, pIX accumulation in tumor cells is generally higher than in healthy cells [19,20]. Irradiation of PpIX preferentially accumulated in tumor cells can result in a significant amount of reactive oxygen species (ROS), such as singlet oxygen, superoxide, hydroxyl radical, and hydrogen peroxide [21,22]. Although 5-ALA is a safe fluorescent contrast agent that has been used for intraoperative margin identification of various types of tumors as well as PDT, there is not much information available on its use with soft tissue sarcomas [18]. A combination between the two approaches, chemotherapeutics and PDT, has already been suggested as an approach for tumor treatment. Numerous in vitro studies have already demonstrated the enhanced therapeutic benefit of PDT and DOX in several cancer entities [23,24,25]. The idea of combining chemotherapy with PDT to kill tumor cells is not new; in fact, the anticipated effects of such a combination were first described three decades ago showing an enhanced tumoricidal effect [26]. Meanwhile, while more recent studies have shown that DOX in combination with PDT has synergistic effects in breast cancer cells [27,28], lung cancer cells [23,29], hematological malignancies [30] as well as various cancer stem cells entities [31], there is little information about ES.

The aim of this study was to investigate effects of 5-ALA-mediated PDT on ES cell lines as a single treatment as well as combined with DOX. We hypothesize that 5-ALA-mediated PDT potentiates the effectiveness of DOX in ES cell lines.

## 2. Materials and Methods

**Figure 1:**
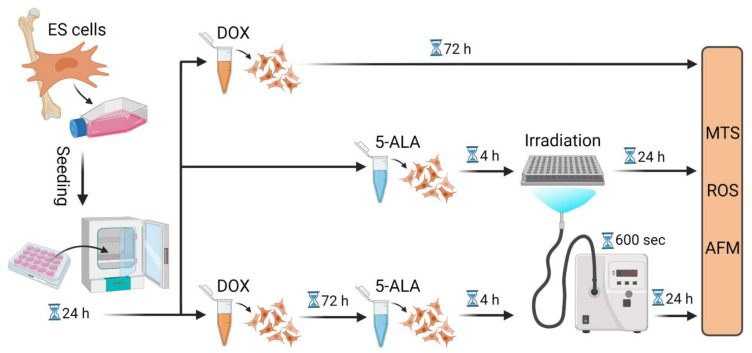
Flow chart of the experimental procedure. Summary of the experimental steps in chronological order, starting from cell seeding to treatment with DOX and/or 5-ALA followed by irradiation. Further measurements were then conducted. Created with BioRender.com. Abbreviations: AFM—atomic force microscope; 5-ALA—5-aminolaevulinic acid; DOX—doxorubicin; ES—Ewing sarcoma; MTS—Cell Proliferation assay; ROS—Cellular Oxidative Stress assay; sec—seconds.

A flow chart of the experimental setup in their chronological order is given in Figure 1. Each experiment was carried out in triplicate and repeated three times.

### 2.1. Cell Lines and Culture

Three human Ewing sarcoma cell lines, namely: RD-ES (HTB-166™; ATCC, Manassas, VA, USA); A-673 (CRL-1598™; ATCC); and TC-71 (ACC 516; Leibniz Institute DSMZ, Braunschweig, Germany) were employed for all experiments. Bone marrow-derived mesenchymal stem cells (MSC) isolated (local ethical approval number: 885/2021BO2) and propagated in culture as previously described [32], were used as a control group. The RD-ES cells were cultured and maintained in RPMI-1640 with L-glutamine (Gibco, Life Technologies, Waltham, MA, USA) media, supplemented with 15% (*v*/*v*) FCS (Biochrom Ltd., Cambridge, UK), and 1% (*v*/*v*) penicillin/streptomycin (Gibco, Life Technologies) while A-673 cells were grown in Dulbecco’s modified Eagle’s medium (Gibco, Life Technologies) supplemented with 10% FCS (Biochrom Ltd.) and 1% (*v*/*v*) penicillin/streptomycin (Gibco, Life Technologies). Iscove’s MDM with L-glutamine (Gibco, Life Technologies) supplemented with 10% (*v*/*v*) FCS and 1% (*v*/*v*) penicillin/streptomycin (Gibco, Life Technologies) was used for TC-71 cells.

### 2.2. PDT Exposure with 5-ALA

For all in vitro experiments, 5-ALA (Sigma-Aldrich, St. Louis, MO, USA) was used as a PS and was dissolved in H_2_O (stock concentration: 50 mM). In preliminary experiments, cells were seeded at different cell densities (0.3–1 × 10^4^ cells/well) in 96-well plates with fresh culture medium and incubated for 24 h at 37 °C in 5% CO_2_ to determine the optimal seeding density and confluency for each cell line. Following 4 h of incubation in the dark, the medium was removed and various concentrations (0.1–0.35 mM) of 5-ALA in serum-free medium were added to the cells. The cells were then exposed to light for a predefined time frame of 600 s. To this end, the IlluminOss System (IlluminOss Medical Inc., East Providence, RI, USA) was employed as the light source. In continuous output mode, it emits blue light with a wavelength of 436 nm (3.8 J/cm^2^). With a distance of 5 cm towards the light source, the cells were evenly illuminated from below in black 96-well plates. Cells that were not exposed to PDT irradiation and/or 5-ALA were used as a control group.

### 2.3. Doxorubicin Treatment

Cells were treated with doxorubicin hydrochloride, a chemotherapeutic agent commonly used to treat ES (DOX HCL; Selleckchem, Houston, TX, USA). Stock solutions (50 mM) in dimethyl sulfoxide (DMSO; Sigma-Aldrich) were prepared and stored at −80 °C. After growing cells in 96-well plates or atomic force microscope (AFM) dishes overnight in fresh culture medium at 37 °C under 5% CO_2_, different DOX concentrations (10–100 nM) in the appropriate media were added to the cells for a 72 h incubation period.

### 2.4. Doxorubicin and 5-ALA PDT Combined Therapy

An amount of 0.3 × 10^4^ RD-ES cells/well and TC-71 cells/well, 0.6 × 10^4^ A-673 cells/well and 0.5 × 10^4^ MSCs per well were seeded in 96-well plates with fresh culture medium and incubated for 24 h at 37 °C in 5% CO_2_. For AFM petri dishes (TPP Techno Plastic Products AG, Trasadingen, Switzerland), RD-ES cells were plated at a density of 2 × 10^4^ cells/plate, A-673 cells and TC-71 were plated at a density of 4 × 10^4^ cells/plate, and MSCs were plated at a density of 2.5 × 10^4^ cells/plate. The cells were then incubated for another 72 h in fresh culture medium containing 15 nM DOX. DOX was removed from the cells prior to PDT irradiation, and FCS-free medium supplemented with 0.15–0.35 mM 5-ALA was added. After 4 h of incubation in the dark, the cells were subjected to PDT for 600 s. Further experiments were carried out 24 h after PDT exposure and 5-ALA, as well as DOX removal.

### 2.5. Cell Viability Assay

The Cell Titer 96^®^ AQueous One Solution Cell Proliferation (MTS) assay (Promega, Mannheim, Germany, Fitchburg) was used to assess cell viability, which is measured colorimetrically due to mitochondrial conversion of yellow 3-(4,5-dimethylthiazol-2-yl)-5-(3-carboxymethoxyphenyl)-2-(4-sulfophenyl)-2H-tetrazolium (MTS) to purple formazan. The amount of formazan is directly correlated with the percentage of viable cells. Following 5-ALA-mediated PDT exposure and DOX treatment, seeded ES cells (for cell density, please see section: “Doxorubicin and 5-ALA PDT combined therapy”) were supplemented with 15 μL of a 2 mg/mL solution of MTS in complete media and incubated for 90 min at 37 °C. At a wavelength of 490 nm, the absorbance was measured using an EL800 microplate reader (BioTek Instruments GmbH, Bad Friedrichshall, Germany).

### 2.6. Reactive Oxygen Species Assay

The activity of reactive oxygen species (ROS) within metabolically active cells was measured using a Cellular ROS Assay Kit ( # ab113851; abcam, Cambridge, UK) following the manufacturer’s protocol. In brief, following 5-ALA PDT with or without DOX treatment, ES cells (for cell density, please see section: “Doxorubicin and 5-ALA PDT combined therapy”) were stained with a 2′,7′-dichlorofluorescein diacetate DCFDA solution (20 μM) and incubated for 45 min at 37 °C and 5% CO_2_ in the dark. Afterwards, the DCFDA reagent was replaced by assay buffer and the resulted fluorescence was measured using a fluorescent microplate reader (GloMAX; Promega, Fitchburg, WI, USA), EM:475 nm/Ex: 500–550 nm.

### 2.7. Cellular Elasticity Assessment

Cellular elasticity measurements were performed as described before [33]. Briefly, measurements were performed in Leibovitz’s L-15 medium without L-glutamine (Merck KGaA, Darmstadt, Germany) using a CellHesion200 (Bruker, Billerica, MA, USA) AFM system, mounted onto an inverted light microscope (AxioObserver D1; Carl Zeiss Microscopy, Jena, Germany). The cells (for cell density, please see section: “Doxorubicin and 5-ALA PDT combined therapy”) were subjected to AFM indentations using a 5 μm spherical tip (model: SAA-SPH-5UM, k = 0.2 N/m; Bruker). The AFM tip was placed above the cells of interest, and three measurements were taken per cell. A total of 30 cells were indented per condition, with three independent replicates performed for each cell line. Indentation curves were sampled at 2 kHz with a 2 nN force trigger. The force–distance curves were processed using the Hertz fit model incorporated in the data processing software (Bruker).

### 2.8. Statistical Analysis

Boxplots and bar diagrams were used to display data graphically. Based on the normality of the data, the Kruskal–Wallis test or analysis of variance (ANOVA) were used to calculate differences between the groups, followed by appropriate post-hoc testing using either the Dunn test or the *t*-test. An alpha adjustment based on a significance level of 0.05 was employed to control type I error in multiple comparisons. To this end, the Bonferroni method for alpha adjustment was employed to control type I error in multiple comparisons adjustment was used to correct raw *p*-values. SPSS statistical software 22 (version 28.0.0.0 (190)); IBM Corp., Armonk, NY) was used for statistical analyses.

## 3. Results

### 3.1. Viability Assessment

#### 3.1.1. DOX and 5-ALA-Mediated PDT Reduce Cell Viability of Human ES Cell Lines

**Figure 2:**
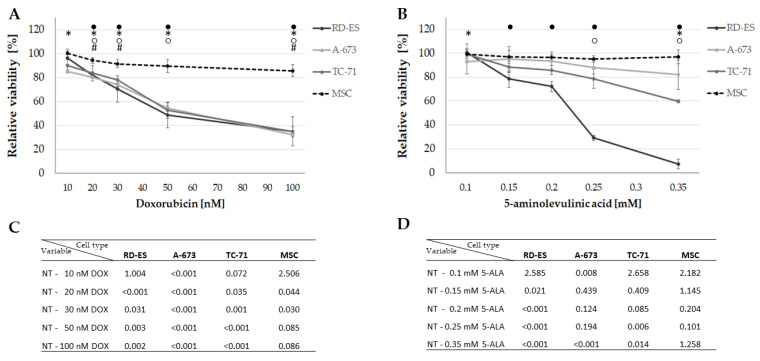
Effect of DOX and 5-ALA PDT on the viability of the ES cell lines and control cells. After 24 h, cells (ES: RD-ES, A-673, TC-71 and MSC) were treated with different concentrations of DOX (**A**) or 5-ALA PDT exposure for 600 sec; (**B**). Negative controls were set to 100% viability and the relative viability was calculated. Mean values were plotted from three independent experiments. Error bars indicate the standard deviations. Statistical significance (*p* < 0.05) compared to NT is indicated by ● for RD-ES, * for A-673, ○ for TC-71 and # for MSC. *p*-values after Bonferroni correction are listed in (**C**) for DOX and (**D**) for 5-ALA PDT. Abbreviations: 5-ALA—5-aminolaevulinic acid; DOX—doxorubicin; ES—Ewing sarcoma; MSC—mesenchymal stem cell; NT—no treatment control; PDT—photodynamic therapy; sec—seconds.

MTS assays were performed to investigate the effect on cellular viability after treatment with DOX and PDT exposure with 5-ALA. The results showed that the cell viability decreased with increasing concentration of DOX (Figure 2A). After treatment with 15 nM DOX, all tumor cell lines showed a reduction in viability to about 80%. The same effect was observed in 5-ALA PDT-exposed cells (Figure 2B), exhibiting cell line-specific sensitivities. A-673 cells were more resistant to 5-ALA PDT, with a viability reduction to around 80% not reached up to a concentration of 0.35 mM, whereas TC-71 cells showed a viability decrease to approximately 80% at a concentration of 0.25 mM 5-ALA. RD-ES cells, on the other hand, showed approximately 80% viability after sensitization with 0.15 mM 5-ALA. The highest doses of 100 nM DOX and 0.35 mM 5-ALA applied to cancer cell lines had no discernible effect on the control group—MSCs (Figure 2A,B). While 15 nM DOX reduced viability of all ES cell lines to about 80%, 100 nM DOX only reduced viability to 85.6% in the control group (MSC), indicating that DOX specifically inhibits viability of ES cell lines. The same applies to 5-ALA, even at the maximum dose of 0.35 mM 5-ALA, a viability of 97% was noted for MSCs. While the treatment with 10 nM DOX showed no significant changes in cell viability, except for A-673 (*p* < 0.001), overall significant changes could be measured for higher concentrations regarding the tumor cell lines (Figure 2A,C). For 5-ALA, a significant viability reduction could only be detected at a maximum concentration of 0.35 mM for all three tumor cell lines (Figure 2B,D). No applied 5-ALA concentration had a significant effect on the control group.

#### 3.1.2. DOX in Combination with 5-ALA-Based PDT Enhances Cell Mortality

**Figure 3:**
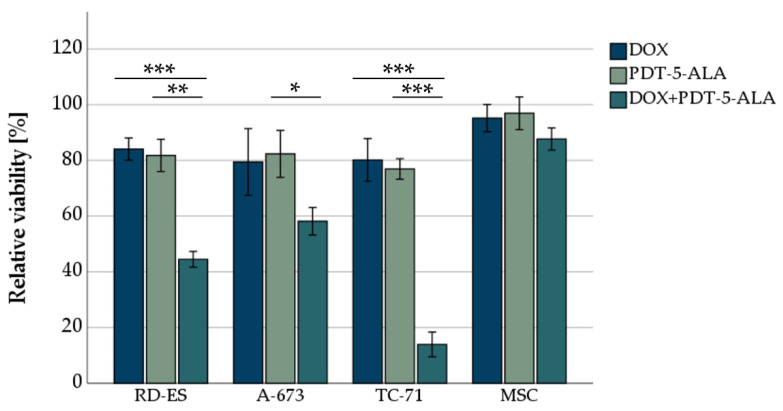
MTS-viability assessment of ES cell lines after DOX treatment and PDT exposure. For DOX, 15 nM was chosen for all three ES cell lines (RD-ES, A-673, TC-71) as well as MSCs. For the photosensitizer 5-ALA, concentrations adapted to the sensitivity of the cell line were chosen (0.15 mM RD-ES, 0.25 mM TC-71, 0.35 mM A-673, and MSCs). Mock-treated control was set to 100% viability. Error bars indicate the standard deviations (* *p* < 0.05, ** *p* < 0.01, *** *p* < 0.001 relative to other treatment). Abbreviations: 5-ALA—5-aminolaevulinic acid; DOX—doxorubicin; ES—Ewing sarcoma; MSC—mesenchymal stem cell; PDT—photodynamic therapy; sec—seconds.

To evaluate the combined effect of DOX and PDT, both treatments were used in combined experiments. Human ES cells and MSCs (as control) were incubated with DOX for 72 h, followed by 5-ALA treatment and irradiation for 600 s. Individual concentrations for each cell line were adjusted to account for DOX and 5-ALA sensitivities (Figure 3). The concentrations were chosen uniformly for comparability so that a viability reduction of about 80% was achieved in all individual treatments (Figure 2). This ensured that, although the cells died partially because of the single treatment, there were still enough cells viable for the effects of the combined treatment to be visible. RD-ES cells exhibited a significant decrease (*p* < 0.01) in viability to 44.5% after combination of 15 nM DOX and 0.15 mM 5-ALA compared to the single treated cells (DOX reduced viability to 84.1%, 5-ALA PDT reduced viability to 81.8%). A-673 cells were more resistant to PDT. After 15 nM DOX and 0.35 mM 5-ALA were applied, a decrease in cell survival to 58.2% was noted, whereas for the single treatment, a reduction to 79.5% for DOX and 82.4% for 5-ALA was observed. The most notable effect was observed in TC-71 cells, where cellular viability was 13.9% after being treated with 15 nM DOX and 0.25 mM 5-ALA post PDT exposure. As a stand-alone therapy, DOX or 5-ALA reduced cellular viability to 80.2% and 76.2%, respectively.

The combination of treatments (DOX and 5-ALA PDT) had no significant effect on MSC cellular viability (87.7% viability after 15 nM DOX and 0.35 mM 5-ALA PDT).

### 3.2. ROS Assessment

#### DOX and 5-ALA-Mediated PDT Induce ROS Production in Human ES Cell Lines

**Figure 4:**
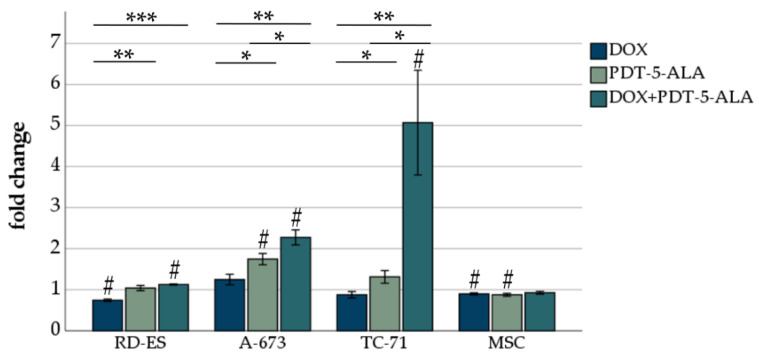
ROS assessment of ES cell lines after DOX treatment and PDT exposure. For DOX, 15 nM was chosen for all three ES cell lines (RD-ES, A-673, TC-71) as well as MSCs. For the photosensitizer 5-ALA, concentrations adapted to the sensitivity of the cell line were chosen (0.15 mM RD-ES, 0.25 mM TC-71, 0.35 mM A-673, and MSCs). Results are presented as mean fold difference of mock-treated control. Error bars indicate the standard deviations (* *p* < 0.05, ** *p* < 0.01, *** *p* < 0.001 relative to other treatment, # *p* < 0.05 compared to untreated control group). Abbreviations: 5-ALA—5-aminolaevulinic acid; DOX—doxorubicin; ES—Ewing sarcoma; MSC—mesenchymal stem cell; PDT—photodynamic therapy; sec—seconds.

As cancer cells are frequently characterized by high cellular oxidative stress and have poor tolerance to oxidative insults [34], we evaluated the ROS production of ES cells following DOX and 5-ALA-mediated PDT treatment. These findings should be inferred in close association with the viability measured by the MTS assay (Figure 3). By taking the TC-71 cells as an example, after the combined treatment, only 20% of these cells were viable (Figure 3). However, these 20% showed a five-fold increase in ROS production (Figure 4), indicating that the remaining 20% of cells were severely damaged. ROS formation decreased significantly (*p* < 0.05) after DOX treatment in RD-ES cells and MSCs compared to an untreated control group (Figure 4). The 5-ALA treatment led to a significant increase (*p* < 0.05) in ROS in A-673 cells, whereas an opposite trend was observed in MSCs. The combined treatment of DOX and 5-ALA PDT showed a significant increase (*p* < 0.05) in all cancer cell lines compared to the untreated control group. No notable change was observed in MSCs. Compared to DOX treatment alone, the combination increased ROS production significantly (*p* < 0.01) in all ES cell lines; the same effect was observed for PDT alone compared to DOX (*p* < 0.05). While for RD-ES cells, no significant difference between the sole 5-ALA PDT irradiation and the combined therapy could be observed, both A-673 cells and TC-71 cells showed a significant increase (*p* < 0.05) in ROS production after the combined treatment. These findings suggest that cells treated with a combination of DOX and 5-ALA PDT exhibited more oxidative stress than mock-treated cells.

### 3.3. Atomic Force Microscopy

#### DOX and 5-ALA-Mediated PDT Increase Stiffness in Human ES Cells Lines

**Figure 5:**
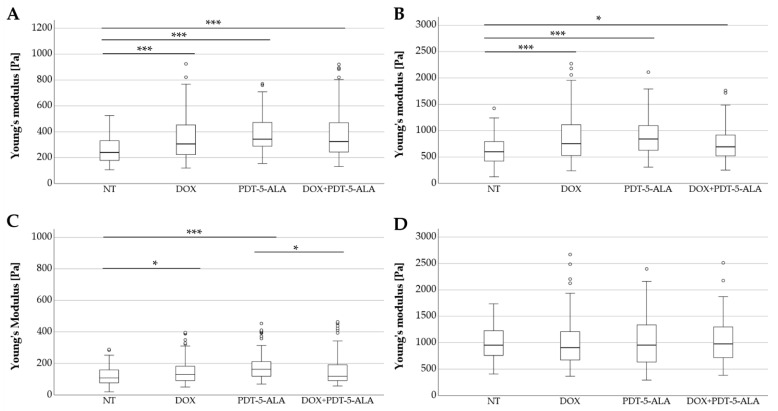
Analysis of Young’s modulus of human ES cell lines and MSC controls. Box plots (medians, minimum, maximum) of the cellular stiffness (Pa) for each cell line is displayed (RD-ES: (**A**) A-673: (**B**) TC-71: (**C**) MSC: (**D**). Outliners are depicted by circles. Statistical significance is indicated by stars over the compared datasets (* *p* < 0.05, *** *p* < 0.001). ES cells treated with DOX, PDT, or in combination were stiffer than their corresponding untreated cells, whereas MSCs serving as controls showed no significant change in elasticity. Abbreviations: 5-ALA—5-aminolaevulinic acid; DOX—doxorubicin; ES—Ewing sarcoma; MSC—mesenchymal stem cell; NT—no treatment control; PDT—photodynamic therapy.

To assess the effect of the treatment approaches on cell stiffness, AFM measurements were performed on all ES cancer cells as well as the control group (MSC). The elastic moduli in the form of the Young’s modulus recorded for the cell lines (RD-ES, A-673, TC-71, and MSC) are depicted in Figure 5A–D. For RD-ES, the cells of each pertaining to each treatment category were significantly stiffer (*p* < 0.001) compared to the untreated control group. Absolute values increased between mock-treated controls and DOX-treated cells by 28% (median: 240 Pa to 307 Pa), by 42% for the 5-ALA-mediated PDT (median: 240 Pa to 243 Pa), respectively, by 35% for the combined approach DOX and 5-ALA PDT (median: 240 Pa to 325 Pa). A similar effect was seen in A-673 cells, where the single treatment with DOX and PDT (*p* < 0.001) as well as the combined approach (*p* < 0.05) showed a significantly altered Young’s moduli profile. Values were increased by 26% for the DOX-treated cells (median: 600 Pa to 754 Pa), by 40% for the PDT treatment (median: 600 Pa to 842 Pa) and 16% for the combined treatment (median: 600 Pa to 694 Pa). Both RD-ES and A-673 cells showed no significant difference between the single treatments and the combined therapy. While for TC-71, treatment with DOX or PDT alone significantly (DOX: *p* < 0.05, PDT: *p* < 0.001) stiffened the cells compared to the untreated group (DOX increased by 19%, median: 109 Pa to 130 Pa; PDT increased by 50%, median: 109 Pa to 163 Pa), no statistically significant change in elasticity was observed between the untreated group and the combined therapy. Interestingly, the cells treated with only PDT had significantly (*p* < 0.05) higher Young’s moduli than the combined treatment approach (median: 163 Pa to 119 Pa). In contrast, the control group (MSC) showed no significant difference in stiffness when compared to non-treated cells or among the treated groups. The median values of the treated MSCs did not differ from the control group by more than 5% (median for NT: 952 Pa, DOX: 905 Pa, PDT-5-ALA: 953 Pa, DOX-PDT-5-ALA: 976 Pa).

In order to shed light on the observed changes in stiffness, we further investigated the effect of DOX and 5-ALA-mediated PDT on ES cells. To this end, changes in cellular cytoskeletal structure were assessed qualitatively using F-actin labeling (Supplementary methods and Figure S1 (Supplementary Materials)). For RD-ES, mock-treated cells showed a typical network of peripheral F-actin (Figure S1A). In general, the cancer cells exhibited fewer short and less organized F-actin fibers (Figure S1A,C,E,G), whereas MSCs showed better-aligned F-actin with well-defined, longer stress fibers (Figure S1B,D,F,H). Actin filaments in the RD-ES cells exhibited a rather randomly tangled network of shorter F-actin strands.

Moreover, a change in the cellular morphology was also noted, with small round cells RD-ES cells observed in the mock-treated group (Figure S1A) compared to enlarged and flattened cells revealed in the cells treated with DOX and 5-ALA-mediated PDT (Figure S1C,E,G).

## 4. Discussion

Current treatment approaches for ES include aggressive local and systemic therapies, which severely limit the quality of life of affected patients. In Europe, the standard chemotherapy for localized ES lesions consists of vincristine, ifosfamide, doxorubicin (DOX), and etoposide [2], with relatively good prognosis. However, for the quarter of patients who present with metastatic disease, survival remains poor, with less than 30% of patients surviving beyond 5 years, regardless of therapy employed [35]. Thus, there is an unmet need for new therapeutic approaches to augment and improve the current standard of care for ES. Although 5-ALA-mediated PDT has been shown to be a viable therapeutic option for the treatment of various types of cancer [18,30,36], its use with soft tissue sarcomas such as ES is not well documented. The goal of this study was to investigate the therapeutic effects of 5-ALA PDT and DOX on ES cell lines, both individually and in combination.

Our results showed that the combination of DOX and 5-ALA PDT efficiently and selectively suppressed the viability of ES cells. All ES cell lines exhibited decreased viability with increasing doses of either DOX or 5-ALA. Moreover, peculiar dose-dependent effects for every cell line have been noted. Overall, these results are in line with existing research [37,38]. All ES cell lines showed similar DOX sensitivities, A-673 and TC-71 exhibited a higher resistance to 5-ALA-mediated PDT compared to RD-ES. While 5-ALA PDT potentiated the efficacy of DOX in all ES cell lines, the most notable effect was observed in TC-71 cells while the MSCs remained viable. Different sensitivities to combined treatment across ES cell lines could also be attributed to different resistances to DOX and different cell fates: apoptosis or senescence [39]. As a p53 non-functional cell line, TC-71 and A-673 cells have been shown to have above-average chemosensitivity, particularly to DOX [40]. Because single treatments only had a minor effect on cell viability, the combined treatment’s high efficacy suggests that it generates the shift inducing cellular death rather than cellular growth arrest (i.e., senescence). While DOX alone may induce senescence in some cancer cells [41], the addition of 5-ALA PDT may activate and trigger cell death, resulting in decreased viability rates similar to those observed in our study. In fact, DOX’s cytotoxicity has already been shown to be enhanced when combined with 5-ALA-mediated PDT [28,30].

Supporting the viability results, an increased oxidative stress after treatment in all cancer cell lines was observed, with the combined therapy having the strongest effect. DOX treatment alone, on the other hand, reduced ROS production in RD-ES and MSCs, despite the fact that the drug is known to work through a variety of mechanisms, including the formation of ROS [42]. This could be explained by the timing of the ROS measurement, which was chosen after incubation due to the subsequent experimental setup with irradiation. While the increased cellular level of ROS improves the killing effect of ROS on cancer cells [43], TC-71 cells showed extremely high ROS production and thus a remarkable decrease in viability, suggesting the metabolism to be highly sensitive to the combined treatment. Significantly lower ROS production was observed in the other cell lines, which could be attributed to defense mechanisms such as ROS-eliminating antioxidants such as glutathione [44].

Since the mechanical fingerprint of the cells is considered to act as a label-free biomarker for metastatic potential [45,46], we investigated the ES elastic properties prior to and post treatment. Our results showed that both individual applications of DOX and 5-ALA PDT as well as their combination significantly reduced cellular stiffness. Cancer cells are less stiff than healthy cells [47], and this peculiar characteristic is believed to be associated with an increased cell invasion and metastatic potential [48]. Moreover, F-actin staining of RD-ES and MSCs (Figure S1) exhibited altered cytoskeletal structure after treatment. Our findings that stiffer cells such as MSCs or treated RD-ES cells have thicker stress fibers are consistent with results from previous studies [49]. Stress fibers have been shown to influence cancer by participating in cellular contractility and thus providing force for cancer motility and migration [50,51]. Furthermore, Grady et al. have already suggested a potential relation between the stability of the cellular F-actin network and changes in the Young’s modulus [47]. The genotoxic drug DOX is known to cause remodeling of actin cytoskeleton architecture; however, its effects are debatable [52]. DOX inhibits the formation of filopodia, which are thin finger-like cell protrusions formed by actin polymerization, in mouse embryonic fibroblasts [53]. Since filopodia protrusions promote the survival of disseminated carcinoma cells [54], DOX-induced attenuation of filopodia formation may contribute to DOX-treated cells’ low viability. Regardless of the treatment method, no cytoskeleton changes were observed in the control cell line—MSC. These findings are consistent with previous research, indicating the therapeutic approach’s safety [55,56].

It has to be noted that light exposure conditions, such as the light source and wavelength, the distance to the target cells, the intensity and duration of the illumination, contribute to the efficacy of PDT and therefore influence the outcome, in particular for in vivo settings. When applied in vivo, PDT is limited by tumor penetration depth as well as oxygen available in the hypoxic core and insufficient PS accumulation [57]. In this current study, our methodological approach is based on clinical circumstances, such as chemotherapy following tumor removal surgery. If PDT was to be applied during tumor resection, it could damage the cancer or, ideally, even induce cell death and thus secure a tumor-free surgical margin. Since the light source used in our experiments is the current version of the clinical product distributed by IlluminOss Medical for its Photodynamic Bone Stabilization system, the application of PDT in a clinical setting is already implementable. Moreover, if PDT was to potentiate the effectiveness of DOX, dose adjustments could mitigate serious side effects, such as cardiotoxicity.

For ES, the combination of 5-ALA-mediated PDT and DOX has a good potential as an innovative therapeutic strategy. However, further research is needed to optimize possible combined treatments and enhance effectiveness of 5-ALA-mediated PDT via in vivo experiments.

Overall, our results show that the 5-ALA PDT exposure exerts selective cytotoxicity for ES cells. A combination approach of 5-ALA-mediated PDT on ES cells after chemotherapy (DOX) has an increased anti-tumorigenic effect, implying that PDT could work in tandem with conventional therapies.

## 5. Conclusions

The present study intended to prove that across multiple ES cell lines, 5-ALA-mediated PDT could successfully and safely be combined with DOX to potentiate the therapeutic effects. While the cancer cell lines exhibited different sensitivities to PDT, the combined therapy with DOX was able to achieve significant impairment of ES cells. Thus, 5-ALA PDT has the potential to be a useful treatment in the future, both on its own and in conjunction with conventional methods. Further research is necessary to determine whether the use of PDT might make it possible to reduce otherwise toxic doses of DOX.

## 6. Study Limitations

Our in vitro study investigated the effect of 5-ALA PDT on several ES cell lines. Since cellular interaction in solid tumors is influenced by a 3D growth pattern, 3D cell culture settings and animal models may come closer to in vivo settings compared to the monolayer (2D) cell culture used in this study.

The distance between the light source and the target cells, as well as the intensity and duration of the illumination, all have an impact on the effect of the light source on the cells. Given all of these variables, the PDT setup for ES would need to be tested and adapted for future in vivo experiments. Our results are, however, in line with previous publications that showed both PDT alone or in combination with drugs such as DOX can be effectively used against various cancer-derived cell lines [23,27,58,59].

Absolute values for AFM stiffness measurements depend on the experimental setup, such as indenter shape and size, indentation velocity and depth, and an analysis adapted to the shape of the tip in the model-fitting employed [60,61], but the tendency within one study remains unaffected.

## Data Availability

All data can be obtained from authors on a reasonable request.

## Supplementary Materials

The following supporting information can be downloaded at: https://www.mdpi.com/article/10.3390/biomedicines10112900/s1. Figure S1. Representative figures of F-actin organization in human ES cells and MSCs after DOX and 5-ALA-mediated PDT.
