# Supplementary material for: 5-Aminolevulinic Acid-Mediated Photodynamic Therapy Potentiates the Effectiveness of Doxorubicin in Ewing Sarcomas
Source: Biomedicines. 2022 Nov 11;10(11):2900. doi: 10.3390/biomedicines10112900 (PMC9687703; doi:10.3390/biomedicines10112900)
Supplement: Supplementary file 1 [file biomedicines-10-02900-s001.zip › biomedicines-2009897-supplementary.pdf]

Online supplement

Methods

Cytoskeleton labelling

Following AFM measurements, RD-ES cells and MSC were fixed for 10 min with 4 % (v/v) paraformaldehyde (PFA, Sigma-Adrich) in PBS at room temperature and washed two times with PBS. Following the manufacturer's instructions, the F-actin cytoskeleton was stained with CellMask™ Green Actin Tracking Stain (#A57243; Thermo Fisher Scientific, Waltham, Massachusetts, USA) and washed three times with PBS. Fluorescent images were recorded using a fluorescence microscope (Leica DMi8 microscope, Leica, Wetzlar, Germany) at a 40-fold magnification.

Results

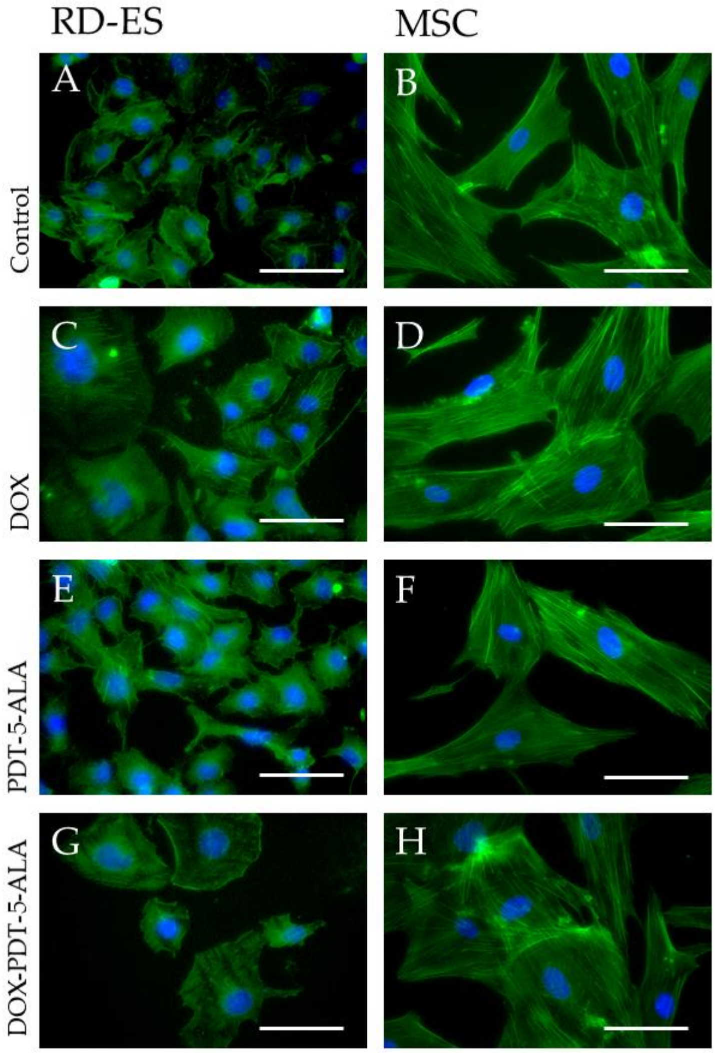

Figure S1. Representative figures of F-actin organization in human ES cells and MSC after DOX and 5-ALA-mediated PDT. Post 5-ALA PDT – DOX treatment RD-ES cell line (**A,C,E,G**) as well as the control cells - MSC (**B,D,F,H**) were subjected to F-actin labelling (green fluorescence). Cell nuclei were stained with DAPI (blue). 40-fold magnification, scale bar (white) represents 200 μM. Abbreviations: 5-ALA – 5-aminolaevulinic acid, DOX – doxorubicin, ES – Ewing sarcoma, MSC- mesenchymal stem cell, PDT – photodynamic therapy, sec – seconds.
